# Effects of Exogenous Hydrogen Sulfide in the Hypothalamic Paraventricular Nucleus on Gastric Function in Rats

**DOI:** 10.3389/fphar.2021.806012

**Published:** 2022-01-13

**Authors:** Chenyu Li, Hongzhao Sun, Yuan Shi, Yan Yu, Xiaofeng Ji, Enguang Li, Xiaofan Zhou, Xiaomeng Liu, Xikang Xue, Haiji Sun

**Affiliations:** ^1^ School of Life Science, Qilu Normal University, Jinan, China; ^2^ Key Laboratory of Animal Resistance, School of Life Science, Shandong Normal University, Jinan, China

**Keywords:** hypothalamic paraventricular nucleus (PVN), hydrogen sulfide, CBS (cystathionine β-lyase), gastric acid secretion, gastric motility

## Abstract

**Background:** Hydrogen sulfide (H_2_S) is a new type of gas neurotransmitter discovered in recent years. It plays an important role in various physiological activities. The hypothalamus paraventricular nucleus (PVN) is an important nucleus that regulates gastric function. This study aimed to clarify the role of H_2_S in the paraventricular nucleus of the hypothalamus on the gastric function of rats.

**Methods:** An immunofluorescence histochemistry double-labelling technique was used to determine whether cystathionine-beta-synthase (CBS) and c-Fos neurons are involved in PVN stress. Through microinjection of different concentrations of NaHS, physiological saline (PS), D-2-Amino-5-phosphonovaleric acid (D-AP5), and pyrrolidine dithiocarbamate (PDTC), we observed gastric motility and gastric acid secretion.

**Results:** c-Fos and CBS co-expressed the most positive neurons after 1 h of restraint and immersion, followed by 3 h, and the least was at 0 h. After injection of different concentrations of NaHS into the PVN, gastric motility and gastric acid secretion in rats were significantly inhibited and promoted, respectively (*p* < 0.01); however, injection of normal saline, D-AP5, and PDTC did not cause any significant change (*p* > 0.05). The suppressive effect of NaHS on gastrointestinal motility and the promotional effect of NaHS on gastric acid secretion could be prevented by D-AP5, a specific N-methyl-D-aspartic acid (NMDA) receptor antagonist, and PDTC, an NF-*κ*B inhibitor.

**Conclusion:** There are neurons co-expressing CBS and c-Fos in the PVN, and the injection of NaHS into the PVN can inhibit gastric motility and promote gastric acid secretion in rats. This effect may be mediated by NMDA receptors and the NF-*κ*B signalling pathway.

## 1 Introduction

Hydrogen sulfide (H_2_S) is a colourless and harmful gas that has the smell of rotten egg. Several studies on H_2_S have been performed in the past Beauchamp et al. (1984), owing to its toxicity. Studies have shown that endogenous H_2_S concentration in the brain is relatively high (50–160 *μ*M) Cheung et al. (2007), indicating that it may have physiological effects. Being the third gaseous signal molecule discovered after NO and CO Łowicka and Bełowski (2007) and since the first report of the physiological role of endogenous H_2_S in mammals, literature on its various biological effects has increased Szabó (2007). Recent studies have revealed that hydrogen sulfide plays an important physiological role in digestive, nervous, and circulatory systems Urszula et al. (2020); Salvatore et al. (2020).

Endogenous H_2_S is mainly synthesised in three ways: first is the catalysed synthesis by cystathionine-beta-synthase (CBS) Kabil et al. (2011); second is the catalysed synthesis by cystathionine-gamma-lyase (CSE) Singh et al. (2009); Whiteman et al. (2009); and third is the catalysed synthesis by 3-mercaptopyruvate sulfurtransferase (3-MPST) Nagahara et al. (1998); Shibuya et al. (2009), which catalyses L-cysteine synthesis of H_2_S. Studies have shown that when CBS inhibitors are injected, CBS levels in the central nervous system decrease Abe and Kimura (1996). CSE is mainly found in the intestines, viscera, muscle epithelial cells, and blood vessels. Since CSE is not found in the brain, the main source of endogenous H_2_S in the central nervous system is the CBS Jimenez (2010).

The hypothalamus is an important part of the diencephalon, which plays an important role in the regulation of food intake, water balance, body temperature, mood, and neuroendocrine Bolborea et al. (2021). Hypothalamic paraventricular nucleus (PVN) is an important neuroendocrine nucleus, and stress response is largely controlled by PVN. PVN plays an important role in coping with digestive diseases and chronic stress Herman et al. (2008). Micromolar amounts of gastrin-17 injected into the PVN of rats can increase the secretion of gastric acid Ohtake and Sakaguchi (1990), and microinjection of thyroid stimulating hormone in the PVN can promote gastric motility and exert its effect through the vagus nerve pathway Morrow et al. (1994). Studies have shown that H_2_S can regulate the release of hypothalamic corticotropin-releasing hormone through the hypothalamus-pituitary-adrenal axis Dello Russo et al. (2000) and plays an important role in regulating gastric function.

N-methyl-D-aspartate receptor (NMDAR) is an ionotropic glutamate receptor. H_2_S and NMDA receptors play a role in reducing the generation of disulfide bonds so that sulfur binds to the sulfhydryl groups of NMDA receptors Aizenman et al. (1989); Greiner et al. (2013). Exogenous injection of glutamate can promote the synthesis of hydrogen sulfide by CBS through the NMDA receptor. NMDA receptor also plays an important role in the neural circuit. In previous experiments, we injected sodium L-glutamate into the suspicious nucleus and dorsal vagus nucleus of rats and found that it inhibited gastric motility and promoted gastric acid secretion in rats. In addition, injection of NaHS also inhibited gastric motility and promoted gastric acid secretion in rats, indicating that it may exert the same effect and may act by stimulating NMDA receptors Sun et al. (2014, 2012). It has been reported for neurons that H_2_S can enhance the activity of NMDA receptors activated by glutamate and promote long-term hippocampal enhancement mediated by NMDA receptors. In addition, H_2_S may cascade regulate NMDA receptors through adenylate cyclase (AC) and develop a dose-dependent trend at an appropriate physiological concentration (10–130 *μ*M) Moshal et al. (2008), indicating that NaHS may regulate gastric function through NMDA receptors.

Nuclear factor kappa-B (NF-*κ*B) is a nuclear transcription factor that controls many inflammation-related genes. Studies have shown that hydrogen sulfide in glial cells is mainly produced by CBS. Inflammatory activation of astrocytes and microglia reduces the expression of CBS, resulting in a reduction in H_2_S production Lee et al. (2009). Injection of NaHS into the lateral ventricle and injection of the NF-*κ*B blocker D-AP5 can reduce neuroinflammation, cognitive impairment, and neuronal damage in rats Gong et al. (2010). By inhibiting the NF-*κ*B signalling pathway, it exerts anti-inflammatory and antioxidant effects to protect gastric mucosa, increase gastric mucosal blood flow Guo et al. (2014), and regulate the expression of cytokines to reduce gastrointestinal inflammation Chen and Liu (2016); Magierowski et al. (2016). Studies have shown that H_2_S may downregulate the NF-*κ*B pathway by reducing the hydrolysis of the disulfide bond of the p65 protein, and the nuclear translocation of p65 protein and NF-*κ*B can cause inflammation Sen et al. (2012), indicating that NaHS may regulate gastric function via NF-*κ*B. This study aimed to clarify the role of exogenous H_2_S in the PVN on gastric function and whether it is regulated by the NMDA receptor and the NF-*κ*B pathway.

## 2 Materials and Methods

### 2.1 Subjects

Male Wistar rats (body weight 270–290 g) were supplied by the Experimental Animal Centre of Shandong University. The rats were raised in an indoor cage with natural rhythm light, 12 h of light every day, and 12 h of darkness. The room temperature was maintained at approximately 25°C, and they were free to eat and drink. After 1 week of rearing to adapt to the environment, the experiment started. Twenty-four hours before the experiment, the rats were starved of feed and water overnight, and other environmental factors remained unchanged. Before the experiment, intraperitoneal injection of 4% chloral hydrate (100 mg/kg body weight) was used for anaesthetisation, and when the whole body muscles were relaxed, the corneal reflex dull, and the breathing slow and uniform, the experiment was conducted. This experiment was approved by the Experimental Animal Ethics Committee of Qilu Normal University, and all experimental protocols were carried out in accordance with the guidelines of the International Pain Research Association, and all procedures were performed according to the guidelines of the International Association for the Study of Pain Zimmermann (1986).

### 2.2 Chemicals

NaHS (2, 4, and 8 nmol), PDTC (2 nmol), D-AP5 (2 nmol), and pontamine sky blue were all purchased from Sigma-Aldrich (St. Louis, MO, United States). NaHS was dissolved in 0.9% normal saline, while the other chemicals were dissolved in dimethyl sulfoxide. Goat serum, anti-CBS rabbit pAb, FITC-conjugated goat anti-rabbit IgG, Cy3 conjugated Goat anti-mouse IgG, and anti-c-Fos mouse pAb were purchased from Servicebio.

### 2.3 CBS and c-Fos Immunohistochemical Fluorescence Double Labelling

The experimental rat was placed in an ether anaesthetised glass bottle, after which, the rat was dizzy and quickly removed from the bottle. A thick rope was used to tie the limbs and teeth of the rat on a homemade wooden board. We choose the restraint water immersion stress RWIS model to activate glial cells Murison and Overmier (1993); Landeira-Fernandez (2004); Glavin et al. (1994); Pare and Glavin (1986). After the rats were awake, they were immersed in a bucket of cold water (21 ± 1°C) prepared in advance, because the xiphoid process of the sternum was required to be flush with the level of cold water. The same time point for each replicate of the experiment was chosen to reduce experimental error. The rats were randomly divided into three groups (*n* = 6) according to different time periods (RWIS 0, 1, 3 h), using 500 ml of 0.01 mol/L phosphate-buffered saline (PBS) and 4% 0.1 mol/L paraformaldehyde (PFA), which were injected into two glass bottles. The RWIS rat was removed and 4% chloral hydrate (100 mg/kg body weight) was injected into the intraperitoneal cavity, and cardiac perfusion was performed. First, the needle of the infusion set was inserted into the aorta from the left ventricle, and then the rat right atrial appendage was cut. The glass bottle containing PBS was immediately opened to wash off the blood in the rat body. After the perfusion was completed, the glass bottle containing PFA was opened. After completion, the rat brain was removed, placed in a container containing PFA solution for fixation, and stored at 4°C for 12 h. Thereafter, the fixed mouse brain was transferred to a 0.1 mol/L 30% sucrose solution for dehydration, and then the hypothalamic area was cut into 30 *μ*m thick coronal sections with a cryostat and stored in 0.01 mol/L PBS.

Multi-well plate was taken and 500 *μ*L PBS was added to each well in advance to wash away the embedding agent and other impurities remaining on the brain slice. Then, the prepared 3% H_2_O_2_ methanol solution was added for 30 min to block endogenous peroxidase activity. Subsequently, the prepared goat serum blocking solution was added for 1 h to increase the permeability of the cell membrane. Next, 500 *μ*L was added to each of the prepared primary antibody working solution (prepared in blocking solution, mouse anti-c-fos with a dilution ratio of 1:750, rabbit anti-CBS with a dilution ratio of 1:500) and incubated overnight (12 h) at 4°C. Thereafter, they were washed with PBST three times the next day for 15 min each time. Finally, 500 *μ*L of the prepared fluorescent secondary antibody working solution was added and reacted for 1 h, followed by washing with PBST three times for 10 min each time to wash off the remaining fluorescent secondary antibody and protect them from light throughout the experiment. The slides were treated with chromium vanadium-gelatin in advance, and the brain slices were attached to the slides and air-dried naturally. Anti-fluorescence quencher was dropped onto a glass slide, covered with a cover glass, and air bubbles were drained in the glass slide. Finally, the sealed fluorescent glass slide was placed under an Olympus Fluorescence confocal microscopy to observe and compare the brain atlas to determine the position of the hypothalamic paraventricular nucleus, observe the CBS and c-Fos-positive neurons number, and take pictures. The expression of c-FOS and CBS in the PVN was counted using Image pro-Plus 6.0 software (Number/0.01 mm^2^).

### 2.4 Studies on the Physiological Functions of NaHS

#### 2.4.1 Experiment Grouping

A series of experiments were performed to confirm the effects of H_2_S on gastrointestinal motility regulation and mechanisms in the PVN. 1) Microinjection of NaHS (0.1 *μ*L, 2 nmol) (*n* = 6) into the PVN on gastric motility; 2) Microinjection of NaHS (0.1 *μ*L, 4 nmol) (*n* = 6) into the PVN on gastric motility; 3) Microinjection of NaHS (0.1 *μ*L, 8 nmol) (*n* = 6) into the PVN on gastric motility; 4) Microinjection of physiological saline (0.1 *μ*L) (*n* = 6) into the PVN on gastric motility as control group; 5) Microinjection of NaHS (0.1 *μ*L, 2 nmol) + PDTC (0.1 *μ*L, 2 nmol) (*n* = 6) into the PVN on gastric motility; 6) Microinjection of NaHS (0.1 *μ*L, 2 nmol) + D-AP5 (0.1 *μ*L, 2 nmol) (*n* = 6) into the PVN on gastric motility; 7) Microinjection of NaHS (0.1 *μ*L, 2 nmol) (*n* = 6) into the PVN on gastric acid secretion; 8) Microinjection of NaHS (0.1 *μ*L, 4 nmol) (*n* = 6) into the PVN on gastric acid secretion; 9) Microinjection of NaHS (0.1 *μ*L, 8 nmol) (*n* = 6) into the PVN on gastric acid secretion; (10) Microinjection of physiological saline (0.1 *μ*L) (*n* = 6) into the PVN on gastric acid secretion; 11) Microinjection of NaHS (0.1 *μ*L, 2 nmol)+PDTC (0.1 *μ*L, 2 nmol) (*n* = 6), influence on gastric acid secretion; 12) Microinjection of NaHS (0.1 *μ*L, 2 nmol)+D-AP5 (0.1 *μ*L, 2 nmol) (*n* = 6), influence on gastric acid secretion.

#### 2.4.2 Microinjection

The head of the rat was fixed on a stereotaxic device (Stoelting 68 ,002, Shenzhen Ruiwode Company, China). According to the provisions of the stereotactic map of the rat brain Buttner-Ennever (1997), two inner ear holes and the incisors of the animal were used for fixation. The height of the dental tray was adjusted at the same time; the height was 3.3 ± 0.4 mm lower than the two ear rods; the bregma and the fontanelle was kept at the same level; and we ensured that error did not exceed 0.3 mm.

The tissue of the head of the rat was removed and the skull was exposed to determine the position of the bregma and posterior bregma, using the three-dimensional coordinates of the bregma as the zero point, according to the PVN centre point in the map (Bregma 1.7 mm backward along the midline, 0.2 mm on both sides, 8.4 mm deep) and rill a small hole with a diameter of about 2 mm into the skull using an electric skull drill. A tip of about 30 *μ*m glass microelectrode was inserted into the brain according to the coordinates. During the experiment, a heating lamp was used to maintain the body temperature of the rats at 37°C.

#### 2.4.3 Recording Gastric Motility

The abdomen of the rat was turned up, a small bite was cut at the fundus of the stomach, and a 5 mm diameter balloon filled with warm water was inserted into the pylorus of the gastric antrum. The airbag was connected to a pressure transducer and BL-420 (Biological Function Experimental System; Chengdu Taimeng Company, China) and record gastric motility.

#### 2.4.4 Collecting Gastric Acid

The oesophageal perfusion method was used to collect the gastric juice. An intubation tube (inner diameter of 2.5 mm) was inserted into the trachea to keep breathing smooth, and then a polyethylene tube (inner diameter of 2 mm) was used for oesophageal intubation with perfuse warm (37°C) normal saline at 2.0 ml/min. In the stomach of the rat, a small opening was made via cut at the junction of the pylorus and duodenum, where there were few blood vessels. Thereafter, a polyethylene tube (inner diameter 3 mm) was inserted into the stomach from the opening, and a dry Petri dish was used to receive gastric juice. The pH value of the gastric juice was compared for three consecutive 10 min before the microinjection to evaluate the changes in gastric acid secretion. The amount of titratable H^+^ was determined by back titration to pH 7.0 using 0.01 N NaOH.

#### 2.4.5 Histological Identification of the Microinjection Site

**FIGURE 1:**
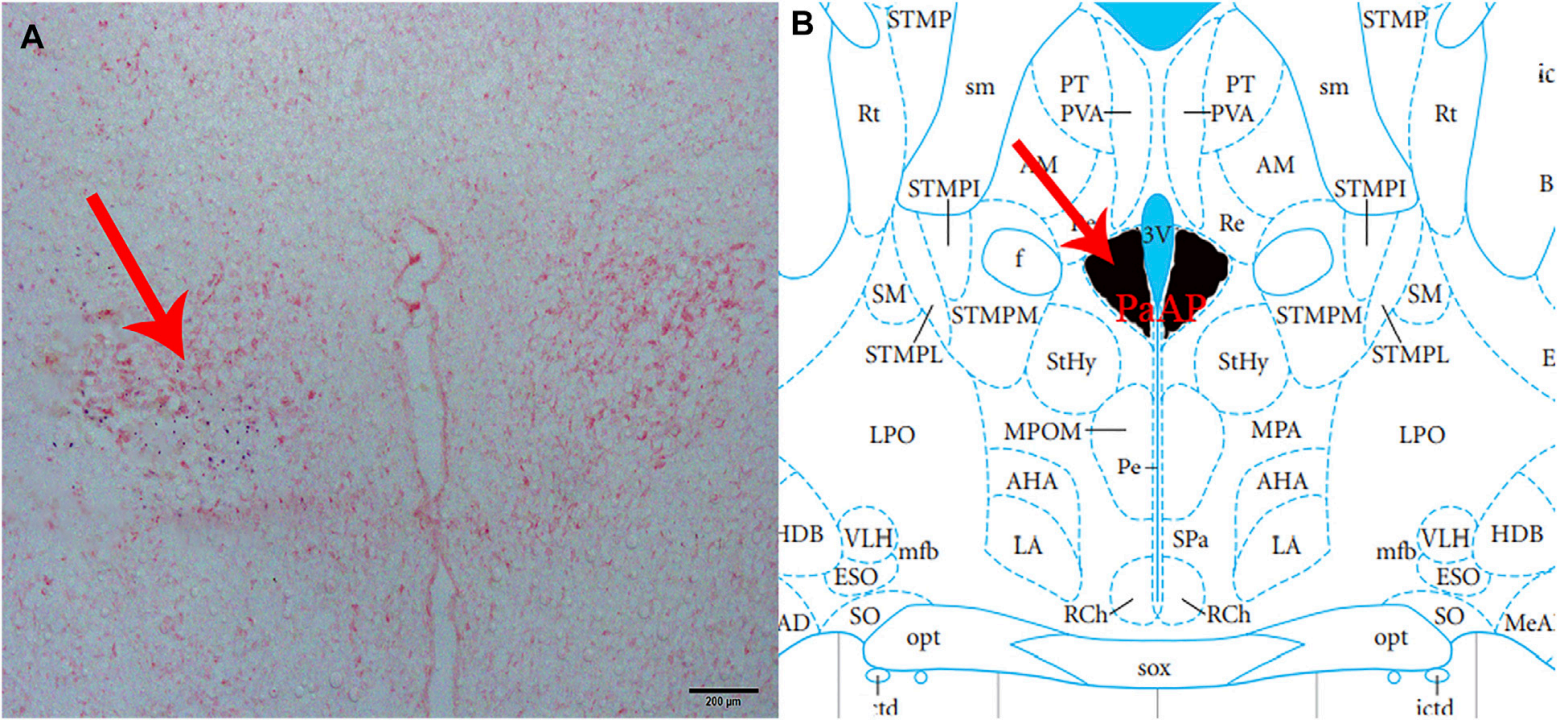
Histological identification of microinjection, the location of the PVN in the brain. **(A)** A brain section stained with neutral red. The blue dot represents injection into the PVN. **(B)** The position of the PVN in the brain atlas.

At the end of each group of the experiments, 2% pontamine sky blue (0.1 *μ*L) was microinjected into the same part of the brain. The rats were then sacrificed by intravenous injection of sodium pentobarbital (80 mg/kg). Subsequently, the blue spot was cut into 16 *μ*m-thick coronal sections with a cryostat and stained with neutral red to confirm that the injection site was located in the PVN (Figure 1). A microscope (Nikon Optiphot, Nikon, Shanghai, China) and a digital camera (Magnafire; Optronics, Goleta, CA, United States) were connected to a computer to take pictures and observe.

#### 2.4.6 Statistical Analysis

Statistics were taken before and after 5 min of microinjection of the average amplitude and average duration of gastric contraction waves. Average gastric motility index (AGMI) is the product of the average amplitude and duration. To determine the changes in gastric motility before and after injection, the inhibition rate was calculated as follows: Inhibition rate (%) = (pre-injection value - post-injection value) × 100%/pre-injection value.

SPSS v25.0 (IBM SPSS Inc, Chicago, Illinois, United States) was used to analyse the results. Student’s t-test or one-way ANOVA was used, followed by the Student-Newman-Keuls test for post-hoc testing. All data are presented as the mean ± standard error. Statistical significance was set at *p* < 0.05.

## 3 Results

### 3.1 CBS and c-Fos Expression in the PVN

**FIGURE 2:**
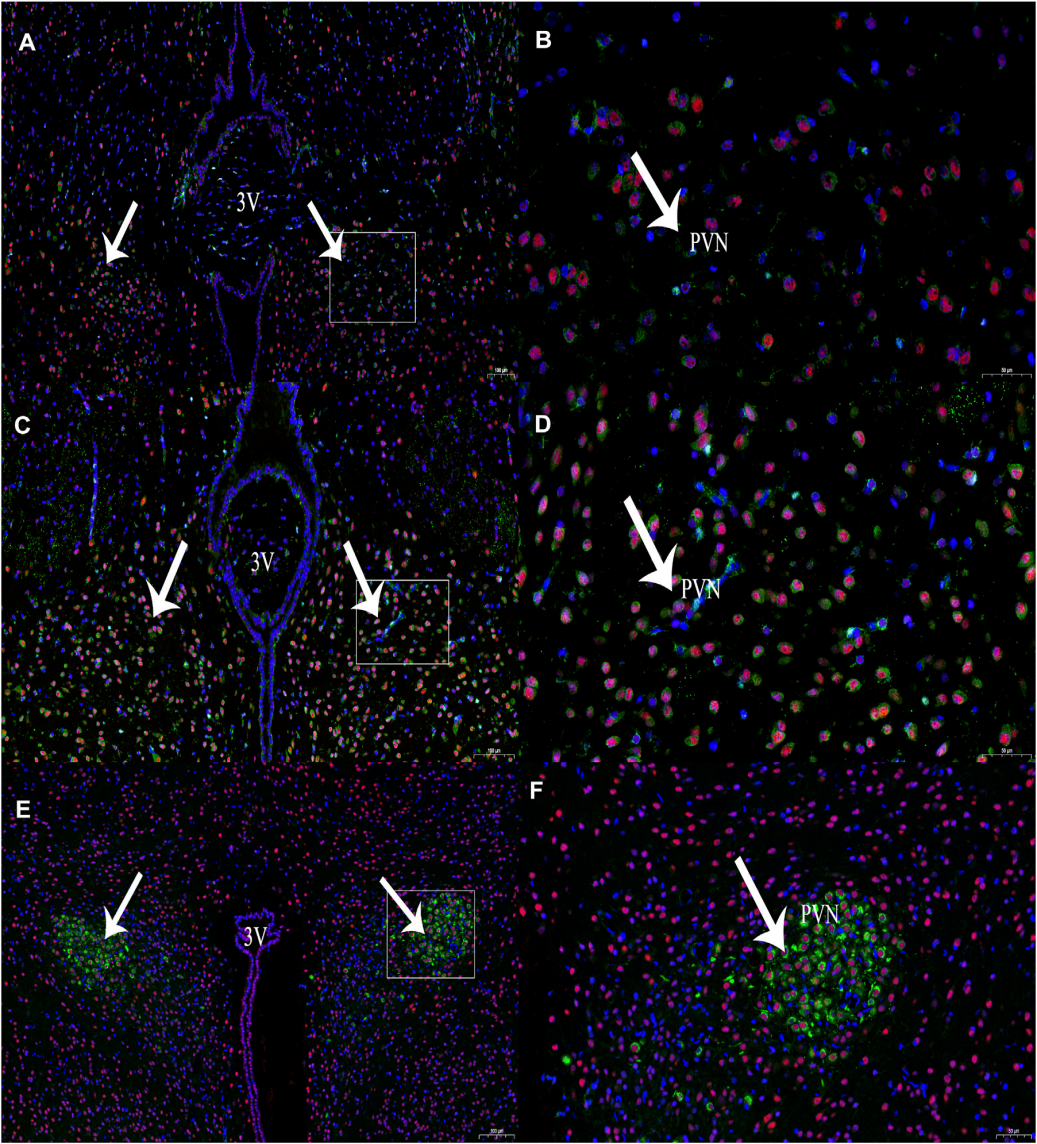
The expression of CBS (green) and c-Fos (red) neurons at different time periods of RWIS. **(A)** The expression of CBS and c-Fos at 0 h of RWIS. **(B)** Enlarged picture of PVN of RWIS for 0 h. **(C)** Expression of CBS and c-Fos for 1 h of RWIS. **(D)** Enlarged picture of PVN of RWIS for 1 h. **(E)** Expression of CBS and c-Fos of restrained immersion for 3 h. **(F)** Enlarged picture of PVN of RWIS for 3 h.

In this experiment, immunohistochemical fluorescence double labelling revealed co-expressions of CBS and c-Fos in the PVN (*n* = 6, Figure 2) at 1 and 3 h compared with those of the control group at 0 h, and the expression of c-Fos protein in the PVN showed an increasing trend and different amplitude changes (Table 1).

**TABLE 1:** Number of neurons co-expressed by CBS and c-Fos in the PVN (Number/0.01 *mm* ^2^, *n* = 6).

Group	Number of CBS neurons containing c-Fos
Control	8.33 ± 0.88
RWIS 1 h	13.83 ± 0.31**
RWIS 3 h	11.50 ± 1.21**

Comparing each RWIS, group with the control group, **p < 0.01.

### 3.2 Effects of NaHS on Gastric Motility

**FIGURE 3:**
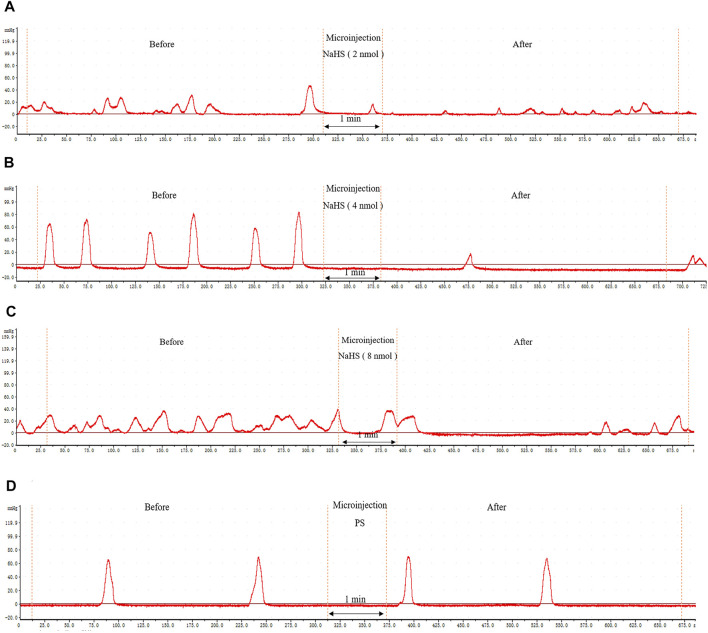
The effect of microinjection of drugs in the PVN on gastric motility in rats. **(A)** The curve of rat gastric movement recorded under the dose of PVN microinjection of 2 nmol NaHS. **(B)** The curve of rat gastric movement recorded under the dose of PVN microinjection of 4 nmol NaHS. **(C)** The curve of rat gastric movement recorded under microinjection of 8 nmol NaHS. **(D)** The curve of rat gastric movement recorded under PVN microinjection of physiological saline.

Microinjection of NaHS (2 nmol, 4 nmol, and 8 nmol, 0.1 *μ*L, *n* = 6) into the rat PVN significantly inhibited gastric motility in rats (Figures 3A–C). Microinjection under the same conditions of physiological saline (0.1 *μ*L, *n* = 6) had no effect on gastric motility in rats (Figure 3D).

**FIGURE 4:**
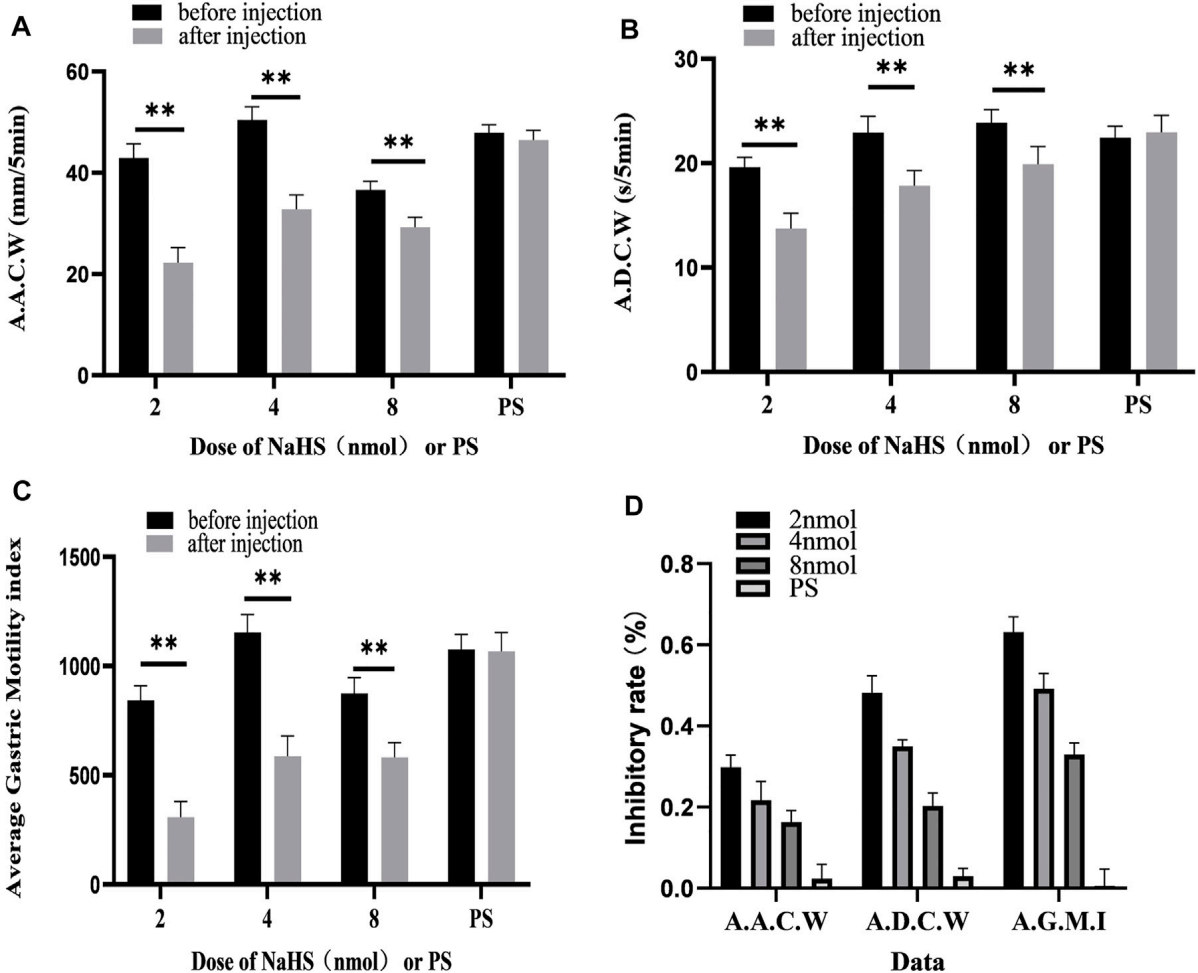
Gastric motility data before and after microinjection of NaHS (2, 4, and 8 nmol) or saline (PS) into the PVN. **(A)** AACW, average amplitude contraction wave. **(B)** ADCW, average duration contraction wave. **(C)** Average gastric motility index. **(D)** Inhibition rate of AACW, ADCW, and average gastric motility index. **p < 0.01, after injection compared with before microinjection.

We also measured, analysed, and compared the data before microinjection and 5 min after microinjection. At the dose of 2 nmol NaHS for PVN microinjection, the average amplitude of contraction waves (AACW) decreased from 42.95 ± 1.14 mm 5 min^−1^ to 22.23 ± 1.23 mm 5 min^−1^ (*p* < 0.01); the average duration of contraction wave (ADCW) decreased from 19.62 ± 0.38 s 5 min^−1^ to 13.74 ± 0.61 s 5 min^−1^ (*p* < 0.01); and the AGMI decreased from 842.51 ± 27.21 to 307.97 ± 29.39 (*p* < 0.01). At the dose of 4 nmol NaHS for PVN microinjection, the AACW decreased from 50.42 ± 1.07 mm 5 min-1 to 32.78 ± 1.17 mm 5 min^−1^ (*p* < 0.01); the ADCW decreased from 22.93 ± 0.63 s 5 min^−1^ to 17.84 ± 0.59 s 5 min^−1^ (*p* < 0.01); and the AGMI decreased from 1,154.85 ± 33.09 to 587.36 ± 37.41(*p* < 0.01). At the dose of 8 nmol NaHS for PVN microinjection, the AACW decreased from 36.60 ± 0.71 mm 5 min^−1^ to 29.21 ± 0.83 mm 5 min^−1^ (*p* < 0.01); and the ADCW decreased from 23.88 ± 0.52 s 5 min^−1^ to 19.92 ± 0.68 s 5 min^−1^ (*p* < 0.01); and the AGMI decreased from 874.55 ± 29.31 to 582.25 ± 27.65 (*p* < 0.01) (Figures 4A–C).

As displayed in Figure 4D, we compared the inhibitory rates of AACW in the 2, 4, and 8 nmol NaHS groups to 48.17%, 35.00%, and 20.33%, respectively. The inhibition rates of the ADCW in the 2, 4, and 8 nmol NaHS groups were 29.83%, 21.67%, and 16.33%, respectively. The inhibition rates of AGMI in the 2, 4, and 8 nmol NaHS groups were 63.17%, 49.17%, and 33.00%, respectively. We found that the inhibition rate of AACW, ADCW, and AGMI of the 8 nmol NaHS group was lower than that of the 4 nmol NaHS group and that of the 2 nmol NaHS group. The results show that the injection of NaHS into the PVN may decrease the inhibitory effect on gastric motility as the dose increases.

### 3.3 PDTC Eliminates the Inhibitory Effect of NaHS on Gastric Motility

**FIGURE 5:**
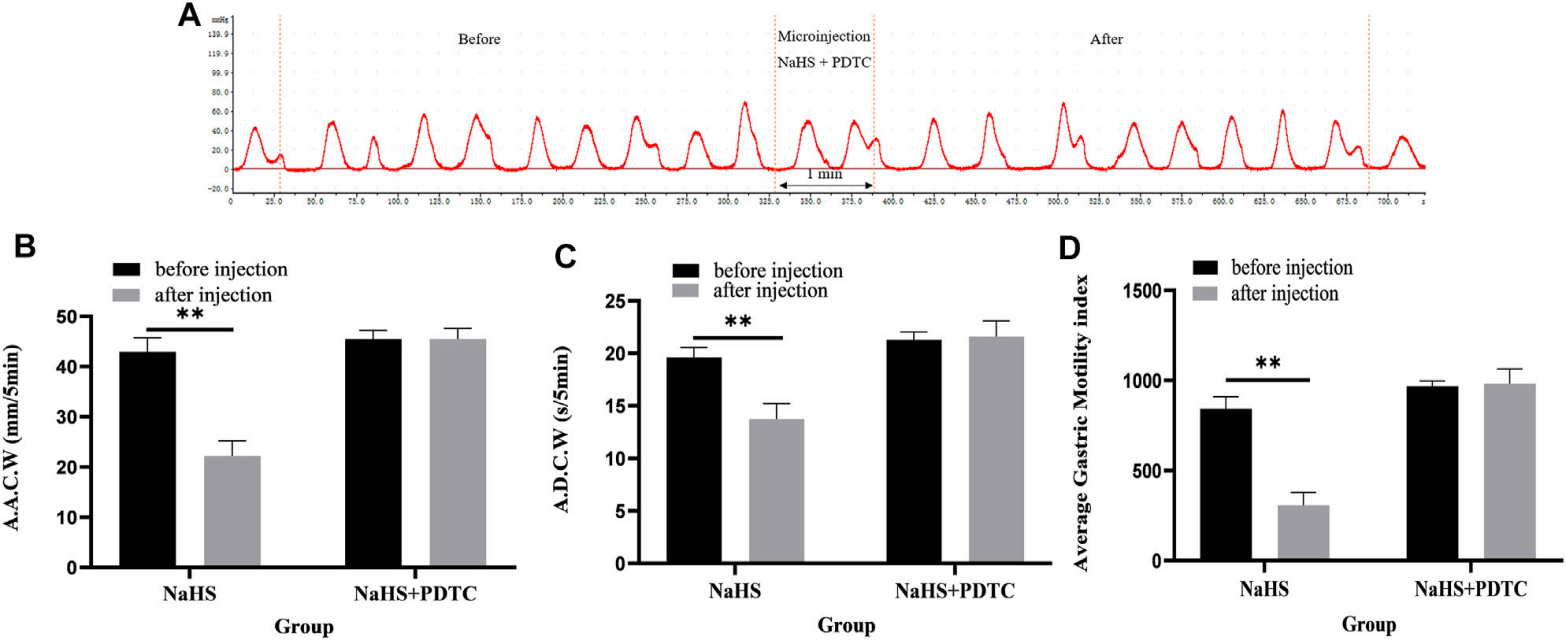
The effect of microinjection of 2 nmol NaHS and 2 nmol NaHS + PDTC into the PVN on gastric motility in rats. **(A)** Curve of gastric motility recorded in rats with 2 nmol NaHS + PDTC microinjection in PVN **(B)** Data of AACW. **(C)** Data of ADCW. **(D)** Data of mean gastric motility index. **p < 0.01, after injection compared with before microinjection.

Microinjection of PDTC and NaHS in the PVN eliminated the inhibitory effect of NaHS on gastric motility (Figure 5A, *n* = 6). As shown in Figures 5B–D, the AACW after the injection of PDTC and NaHS changed from 45.49 ± 0.71 mm 5 min^−1^ to 45.50 ± 0.86 mm 5 min^−1^ (*p* > 0.05). The ADCW changed from 21.29 ± 0.30 s 5 min^−1^ to 21.59 ± 0.61 s 5 min^−1^ (*p* > 0.05), and the AGMI changed from 967.92 ± 12.07 to 982.02 ± 32.96 (*p* > 0.05). These results indicate that NaHS can regulate gastric motility through the NF-*κ*B signalling pathway.

### 3.4 D-AP5 Eliminates the Inhibitory Effect of NaHS on Gastric Motility

**FIGURE 6:**
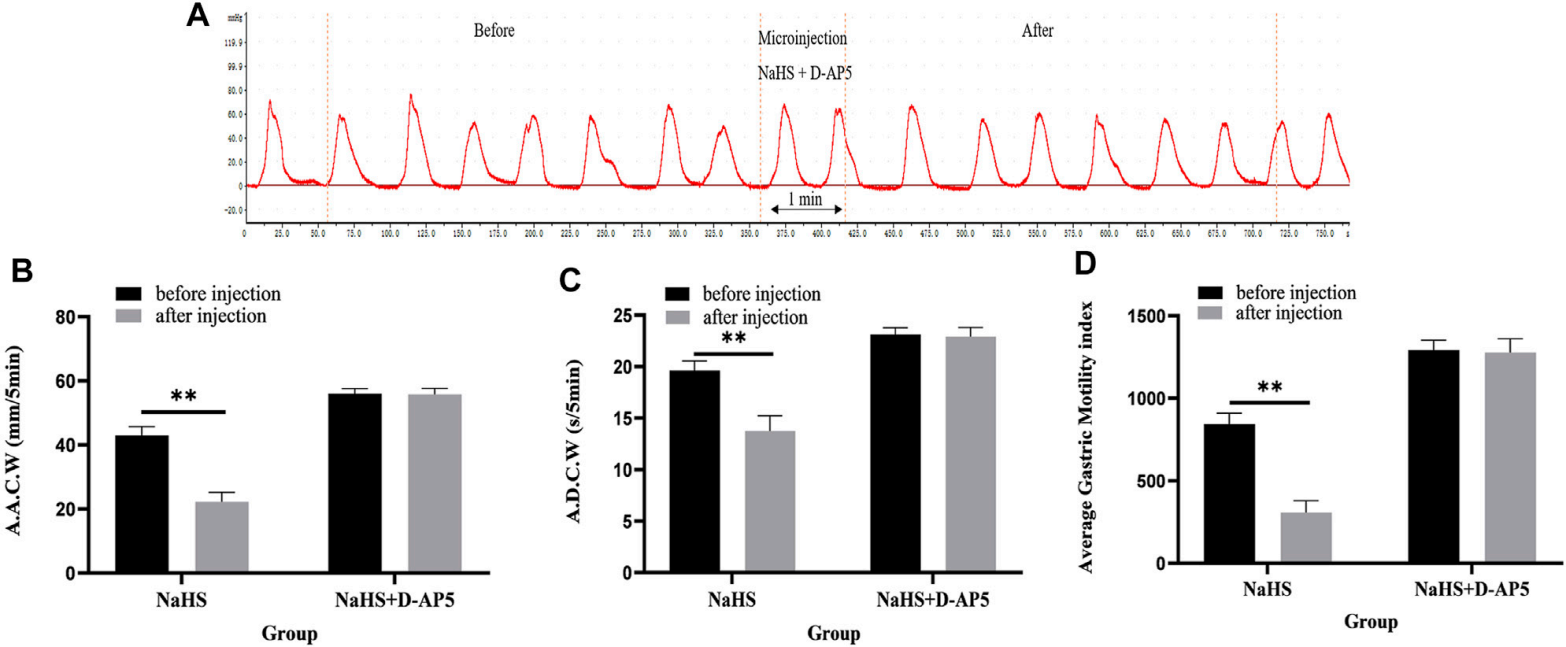
The effect of microinjection of 2 nmol NaHS and 2 nmol NaHS + D-AP5 into the PVN on gastric motility in rats. **(A)** Curve of gastric motility recorded in rats with 2 nmol NaHS + D-AP5 microinjection in PVN **(B)** Data of AACW. **(C)** Data of ADCW. **(D)** Data of mean gastric motility index. **p < 0.01, after injection compared with before microinjection.

Microinjection of D-AP5 and NaHS in the PVN eliminated the inhibitory effect of NaHS on gastric motility (Figure 6A, *n* = 6). As shown in Figures 6B–D, the AACW after the injection of PDTC and NaHS changed from 55.95 ± 0.71 mm 5 min^−1^ to 55.74 ± 0.85 mm 5 min^−1^ (*p* > 0.05). The ADCW was changed from 23.11 ± 0.3 s 5 min^−1^ to 22.91 ± 0.39 s 5 min^−1^ (*p* > 0.05), and the AGMI changed from 1,293.34 ± 26.22 to 1,277.79 ± 37.08 (*p* > 0.05). These results indicate that NaHS can regulate gastric motility by acting on NMDA receptors.

### 3.5 The Effect of NaHS on Gastric Acid Secretion

**FIGURE 7:**
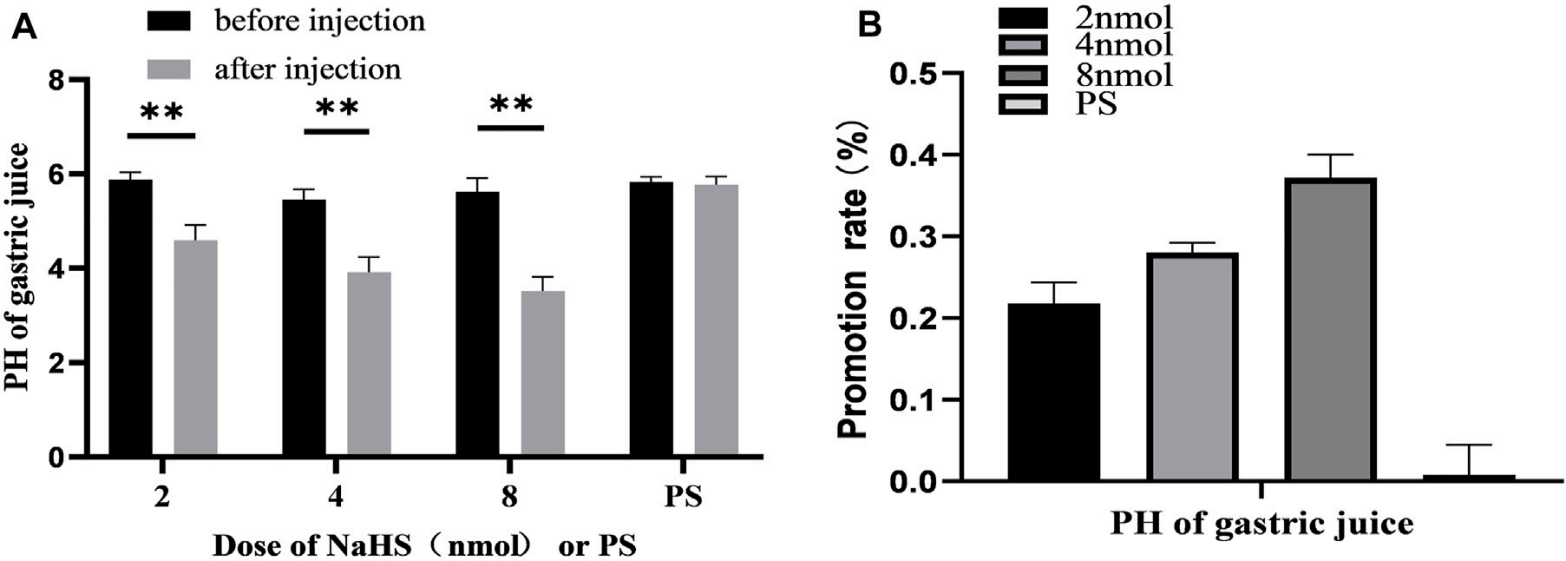
**(A)** The effect of PVN microinjection of 2, 4, 8 nmol NaHS and normal saline on gastric acid secretion. **(B)** The promotion rate of 2, 4, 8 nmol NaHS and PS on gastric acid secretion **p < 0.01, after injection compared with before microinjection.

After microinjection of NaHS (2 nmol, 4 nmol, and 8 nmol, 0.1 *μ*L, *n* = 6) into the PVN, it was revealed that the secretion of gastric acid in the rat was significantly promoted. Under the same conditions, microinjection of PS (0.1 *μ*L, *n* = 6) had no effect on gastric acid secretion in rats (Figure 7A).

The data of 30 min before and after microinjection were analysed and compared. At the dose of 2 nmol NaHS microinjection in PVN, the pH value of gastric acid decreased from 5.88 ± 0.06 to 4.59 ± 0.13 (*p* < 0.01); at the dose of 4 nmol NaHS microinjection in PVN, the pH value of gastric acid decreased from 5.46 ± 0.08 to 3.91 ± 0.13 (*p* < 0.01); and at the dose of 8 nmol NaHS microinjection in PVN, the pH value of gastric acid decreased from 5.62 ± 0.12 to 3.52 ± 0.12 (*p* < 0.01).

As shown in Figure 7B, the promotion rate of gastric acid secretion in the 2, 4, and 8 nmol NaHS groups was 21.83%, 28.00%, and 37.17%, respectively. We found that the promotion rate of gastric acid secretion in the 2 nmol NaHS group was lower than that in the 4 nmol NaHS group and that in the 8 nmol NaHS group. These results indicate that the injection of NaHS into the PVN may enhance the promotion of gastric acid secretion as the dose increases.

### 3.6 PDTC Eliminates the Promoting Effect of NaHS on Gastric Acid Secretion

**FIGURE 8:**
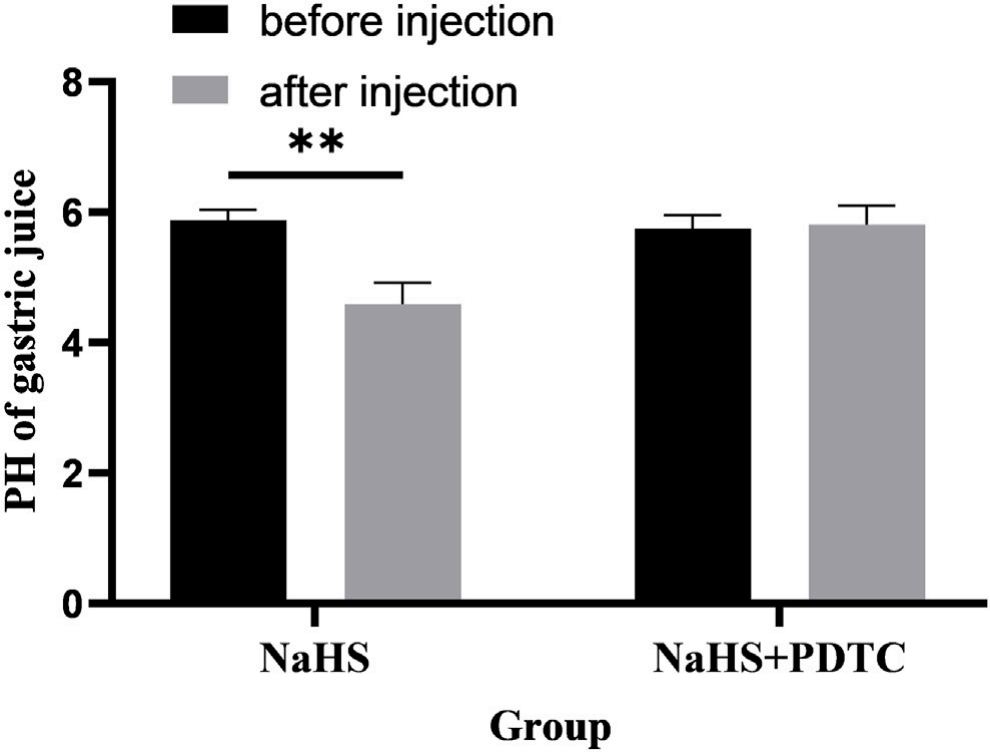
The effect of microinjection of 2 nmol NaHS and 2 nmol NaHS + PDTC into the PVN on gastric acid secretion in rats. **p < 0.01, after injection compared with before microinjection.

Microinjection of PDTC (an NF-*κ*B inhibitor) and NaHS in PVN eliminated the promoting effect of NaHS on gastric acid secretion (Figure 8, *n* = 6). After injection of PDTC and NaHS, the pH value of gastric acid changed from 5.75 ± 0.09 to 5.81 ± 0.12 (*p* > 0.05). The pH value of gastric acid did not change significantly after PVN microinjection of PDTC + NaHS, indicating that NaHS may regulate the secretion of gastric acid through the NF-*κ*B signalling pathway.

### 3.7 D-AP5 Eliminates the Promoting Effect of NaHS on Gastric Acid Secretion

**FIGURE 9:**
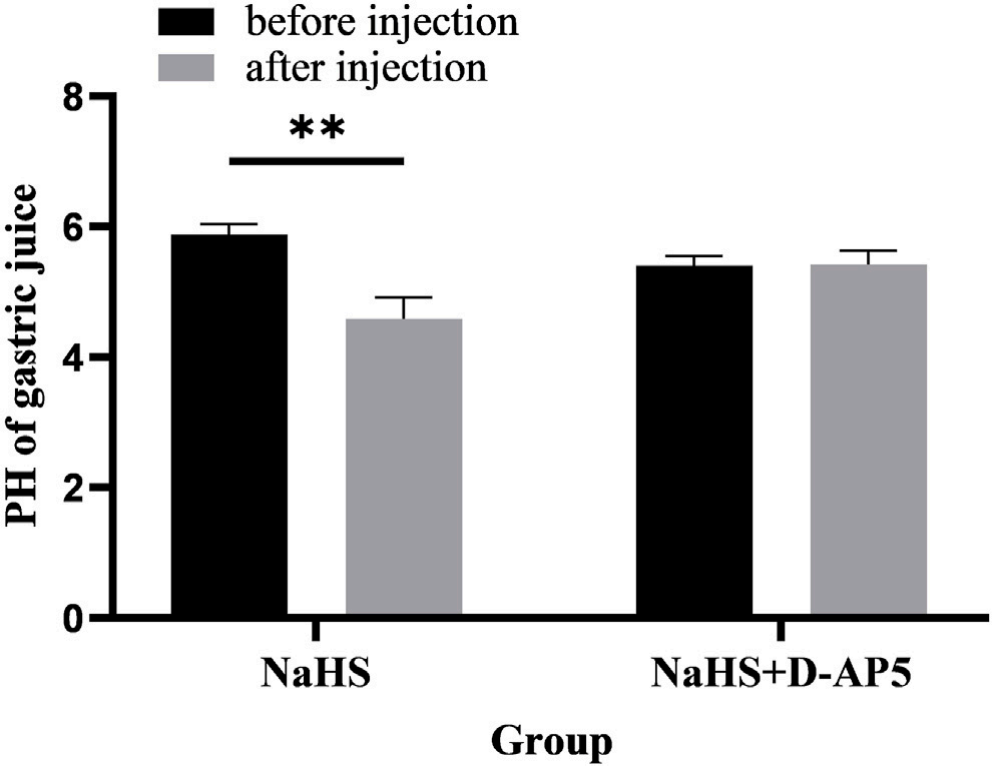
The effect of microinjection of 2 nmol NaHS and 2 nmol NaHS + D-AP5 into the PVN on gastric acid secretion in rats. **p < 0.01, after injection compared with before microinjection.

Microinjection of D-AP5 and NaHS in the PVN eliminated the promoting effect of NaHS on gastric acid secretion (Figure 9, *n* = 6). After injection of D-AP5 and NaHS, the pH value of gastric acid changed from 5.40 ± 0.06 to 5.42 ± 0.09 (*p* > 0.05). There was no significant change in the pH value of gastric acid after PVN microinjection of D-AP5 + NaHS, indicating that NaHS may regulate the secretion of gastric acid by acting on NMDA receptors.

## 4 Discussion

NaHS aqueous solution can be used as a water-soluble donor for quickly production of large amount of H_2_S to simulate the concentration of H_2_S in the physiological environment of cells Sparatore et al. (2011); Lee et al. (2010); Gu and Zhu (2011). H_2_S is a lipophilic molecule that can diffuse freely across the cell membrane Mathai et al. (2009). Therefore, we used NaHS as the H_2_S donor in our experiments.

In this experiment, we first investigated whether there are neurons co-expression of CBS and c-Fos in the PVN. The results showed that in RWIS 1 h rat PVN, CBS and c-Fos co-expressed the most neurons, followed by 3 h, and then 0 h, which was the least. The presence of CBS in PVN indicates that H_2_S in PVN has physiological functions. The experimental results show that exogenous H_2_S in the PVN participates in the regulation of gastric motility and gastric acid secretion in rats under anaesthesia.

Endogenous H_2_S in the hypothalamus can release corticotropin-releasing hormone, induce long-term hippocampal enhancement, and regulate blood pressure and heart rate through K^+^-ATP channels Dawe et al. (2008). Studies have shown that low concentrations of H_2_S may have a protective effect, while high concentrations of H_2_S can damage cells and destroy cell membranes Hegde and Bhatia (2011); Yuan et al. (2016). Determining the appropriate dose of H_2_S is crucial for exploring the development of the therapeutic effect of H_2_S Zheng et al. (2018). Studies have revealed that endogenous H_2_S cooperates with NO. This indicates that endogenous H_2_S acts as a neuromodulator in the brain and can act on smooth muscle cells of the ileum, causing smooth muscle relaxation through an unclear mechanism Kimura (2000); Kimura (2010). These experiment results show that low concentrations of H_2_S significantly increased vasodilation and smooth muscle relaxation induced by the NO donor sodium nitroprusside and inhibited gastric motility. At the same time, H_2_S can also increase the level of glutathione to eliminate oxygen free radicals to resist oxidation and fight inflammation and anti-apoptosis Kimura (2014). Recent studies have also identified that H_2_S may act through the p38-MAPK, PKA/cAMP signalling pathwayPanthi et al. (2018).

In this experiment, we found that after injection of different concentrations of NaHS into the PVN in rats, the AACW, ADCW, and AGMI of the rats were significantly lower than those in the control group. Compared with the control group, the injection of different concentrations of NaHS significantly reduced the pH value of the rat gastric juice and promoted the secretion of gastric acid in rats. KATOH also reported that injection of oxytocin into the PVN inhibited gastric acid secretion in rats and the effect was blocked by vagotomy and atropine Katoh and Ohtake (1991). Miinnikes et al. injected CRF into the paraventricular nucleus of the hypothalamus and found a dose-dependent inhibition of gastric motility, and vagotomy and atropine could eliminate the inhibitory effect of gastric motility Mönnikes et al. (1992). DMV plays an important role in regulating gastric function (such as gastric acid secretion and gastric motility) Shapiro and Miselis (1985); Berthoud et al. (1991). The vagus nerve complex (DMV) can transmit information through the vagus nerve to the stomach, and there is no direct connection between the PVN and the stomach; therefore, we hypothesised that the neurons in the PVN are projected to the DMV and are related to cholinergic neurons innervating the gastric smooth muscle in DMV Lu et al. (2011). We believe that the inhibition of gastric motility may be caused by the inhibitory neurotransmitter VIP or NO released by the vagus nerve to inhibit gastric motility in rats. H_2_S plays a regulatory role in the gastrointestinal tract through its excitatory effect on gastrointestinal nervesv Matsunami et al. (2012); Ise et al. (2011) and regulates intestinal contractility Teague et al. (2002). At physiological concentrations, H_2_S can induce relaxation of the respiratory tract, blood vessels, intestines, and bladder smooth muscle relaxation, which may be mediated by the hyperpolarisation of the membrane potential Zhao et al. (2009). In addition, after the gastric nervous system is stimulated, acetylcholine may be released to bind to receptors in parietal cells, pump out H^+^, and promote gastric acid secretion. This study also revealed that oral administration of NaHS in rats inhibited the contraction of jejunum and ileum smooth muscle and showed a dose-dependent inhibition Nagao et al. (2011), indicating that H_2_S can reversibly inhibit the contraction of ileum spontaneous and cholinergic smooth muscle of rat and can through cholinergic agonists cause changes in the contraction frequency.

In addition, this experiment reveals that the first injection of NMDA receptor blocker D-AP5 and then injection of NaHS eliminated the regulation of gastric function by NaHS. In the central nervous system, H_2_S has been proven to be produced by CBS and acts as a modulator of the long-term potentiation of NMDA receptors. The activity of NMDA receptors increases with a decrease in disulfide bonds, and H_2_S may also enhance the activation of NMDA receptors by reducing cysteine sulfur hydration Zhang and Bian (2014). H_2_S in the spinal cord can also cause hyperalgesia through NMDA receptors, indicating that painful stimulation could also activate NMDA receptors Zhao et al. (2016). Studies have revealed that H_2_S can activate NMDA receptors in the amygdala of rats, and blocking the NMDA receptors in the amygdala eliminates the effect of H_2_S to enhance the long-term potentiation of the amygdala, showing that H_2_S can promote the amygdala to contain glutamate functions as an The L-cys required by astrocytes to synthesise glutathione needs to be synthesised by CBS and is related to NMDA receptor-mediated response regulation McBean (2012, 2017); Huang and Moore (2015). NMDA receptor to regulate emotional memory Wang et al. (2015). H_2_S can also reduce oxidative stress in the body by activating NMDA receptors, avoiding damage from oxygen free radicals and reducing neuronal damage Kamat et al. (2015).

NaHS injection can reduce the symptoms of hepatitis in rats through NMDA receptor anti-inflammatory and antioxidant effects Kwon et al. (2019). PVN regulates blood pressure and heart rate in rats through NMDA receptors Sharma et al. (2021). Excessive H_2_S increases the concentration of glutamate beyond the normal physiological concentration range and causes neurotoxicity García-Bereguiaín et al. (2008). Therefore, we believe that H_2_S can regulate gastric function by reducing oxidative stress through NMDA receptors, but it should be within the normal physiological concentration range.

Furthermore, this experiment revealed that the first injection of the NF-*κ*B blocker PDTC and the subsequent injection of NaHS eliminated the regulation of gastric function by NaHS. Inflammation is a complex process that is coordinated by pro-inflammatory and anti-inflammatory processes. Several evidence show that H_2_S plays a role in inflammatory process Kabil et al. (2014); Zhi et al. (2007); Li et al. (2005); Collin et al. (2005). When inflammation occurs, microglia and astrocytes are activated. Inflammatory stimulation by microglia and astrocytes reduces the expression of CBS. The lack of H_2_S synthesis can lead to some gastrointestinal diseases, and H_2_S can treat neuroinflammation by downregulating the NF-*κ*B pathway Hu et al. (2011); Mrak (2009); Gong et al. (2011). In the present experiment, it was shown that the injection of S-diclofenac and HS-NAP (the donor of hydrogen sulfide) inhibited the NF-*κ*B pathway and reduced the expression of c-Fos in the brain by reducing the nuclear translocation of NF-*κ*B and reducing NF–*κ*B DNA bindings to inhibit the activity of the NF-*κ*B pathway Li et al. (2007); Ravinder et al. (2015). Studies have shown that NaHS has anti-inflammatory properties. Low-dose NaHS reduces NF-*κ*B activity, while high-dose NaHS increases the synthesis of pro-inflammatory factors and NF-*κ*B activity Whiteman et al. (2010); Jeddi et al. (2020); Yin et al. (2013). The anti-inflammatory properties of H_2_S are mediated by improving the protective effect of gastric mucosa and controlling gastric acid secretion, reducing the adhesion of white blood cells to the capillary endothelium of gastric mucosa, and inhibiting the synthesis of pro-inflammatory cytokines Aboubakr et al. (2013). The anti-inflammatory effect of H_2_S is limited to the central nervous system (CNS)-derived glial cells, downregulating a series of inflammatory responses triggered by the activation of primary neurons and subsequent release of inflammatory neuropeptides. The H_2_S donor significantly reduced the inflammatory response of the colon and rectum and relaxed the smooth muscle of the intestine, which significantly reduced the symptoms of hyperalgesia Wallace (2010). Our study showed that H_2_S may be a potential method for the treatment of stress gastric ulcers. In short, we hypothesise that different concentrations of H_2_S will produce outcomes that are the opposite of these experimental results and the influence of H_2_S on the inflammatory mechanism also depends to a large extent on the choice of H_2_S donor. Physiological concentrations of H_2_S can regulate the inflammatory process through the NF-*κ*B pathway and accelerate the healing of gastric mucosa by downregulating the inflammatory response.

**FIGURE 10:**
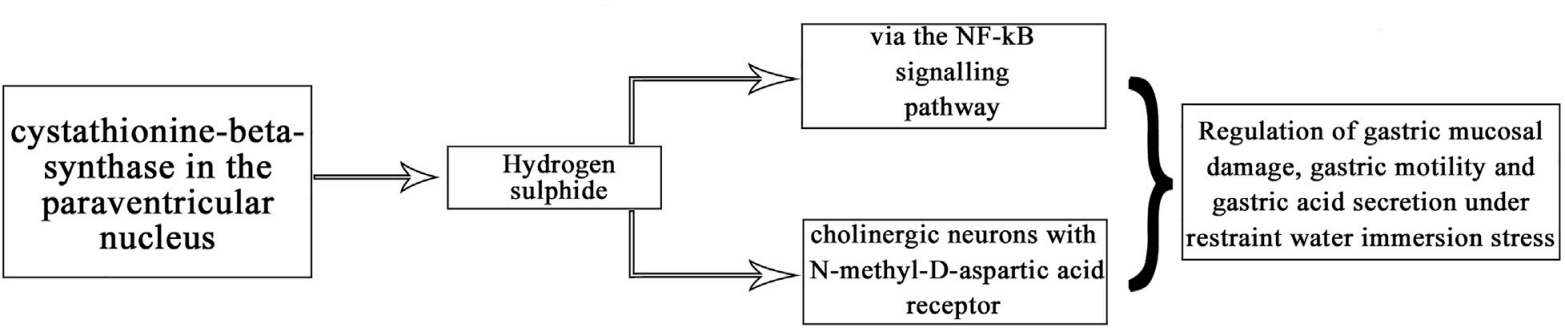
Relationship between H_2_S and NMDA receptor/NF-*κ*B pathway.

In summary, our experiments revealed that CBS neurons in the PVN affect gastric function and exogenous hydrogen sulfide in the PVN significantly inhibit gastric motility and promote gastric acid secretion in rats. This may be due to NMDA receptors and downregulation of the NF-*κ*B signalling pathway (Figure 10). This is the first time that hydrogen sulfide in the PVN may regulate gastric function. Corresponding receptor (or channel) blockers can be designed for use as clinical therapeutic agents, or drug complexes can be synthesised to modulate the release of H_2_S to reduce or prevent stress-related gastric mucosal damage.

## Data Availability

All datasets presented in this study are included in the article/Supplementary Material.
